# Supplementary material for: Injectable human recombinant collagen matrices limit adverse remodeling and improve cardiac function after myocardial infarction
Source: Nat Commun. 2019 Oct 25;10:4866. doi: 10.1038/s41467-019-12748-8 (PMC6814728; doi:10.1038/s41467-019-12748-8)
Supplement: Supplementary file 1 — Supplementary Information [file 41467_2019_12748_MOESM1_ESM.pdf]

Supplementary information for

Injectable human recombinant collagen matrices limit adverse remodeling and improve cardiac function after myocardial infarction

by

Sarah McLaughlin[1,2,7], Brian McNeill[1,7], James Podrebarac[1,2], Katsuhiro Hosoyama[1], Veronika Sedlakova[1], Gregory Cron[3], David Smyth[4], Richard Seymour[1], Keshav Goel[1], Wenbin Liang[2,5], Katey J. Rayner[6,7], Marc Ruel[1,2], Erik J. Suuronen[1,2,8], and Emilio I. Alarcon[1,7,8]

[1]BioEngineering and Therapeutic Solutions (BEaTS), Division of Cardiac Surgery, University of Ottawa Heart Institute, 40 Ruskin street, Ottawa, ON, Canada, K1Y4W7. [2]Department of Cellular & Molecular Medicine, University of Ottawa, Ottawa, 451 Smyth Road, Ottawa, ON, Canada, K1H8M5. [3]Department of Radiology, Faculty of Medicine, University of Ottawa, 501 Smyth Road, Ottawa, ON, Canada K1H8L6. [4]Cardiac Function Laboratory, University of Ottawa Heart Institute, 40 Ruskin street, Ottawa, ON, Canada, K1Y4W7. [5]Cardiac Electrophysiology Lab, University of Ottawa Heart Institute, 40 Ruskin street, Ottawa, ON, Canada, K1Y4W7. [6]Cardiometabolic microRNA Laboratory, University of Ottawa Heart Institute, 40 Ruskin street, Ottawa, ON, Canada, K1Y4W7. [7]Department of Biochemistry, Microbiology, and Immunology, University of Ottawa, 451 Smyth Road, Ottawa, ON, Canada, K1H8M5. [7]These authors contributed equally: Sarah McLaughlin, Brian McNeill. [8]These authors jointly supervised this work: Erik J. Suuronen, Emilio I. Alarcon. Correspondence and request for materials should be addressed to E.J.S. (email: esuuronen@ottawaheart.ca) and E.I.A. (email: ealarcon@ottawaheart.ca)

INDEX

**Page S1**	This page
**Page S1-2**	Index
**Page S3**	**Figure 1.** Representative images depicting the intramyocardial injections for the rHCI matrix.
**Page S4**	**Figure 2.** Effect of chondroitin on collagen matrices physical properties.
**Page S5**	**Figure 3.** Histological examination of rHC hydrogel-treated MI mouse hearts at 2 hours post-injection.
**Page S6**	**Figure 4.** Changes in left ventricle ejection fraction (LVEF%) for collagen matrices and non-crosslinked collagen solutions.
**Page S7**	**Figure 5.** Change in systolic volume and end-diastolic volume for the different treatment groups.
**Page S8**	**Figure 6.** Flow cytometry analysis of mouse blood at 2 days post-treatment. Live single cells were sorted into leukocyte subsets based on the expression of CD45, CD11b, Ly6G, CD3, B220, F480 and Ly6C.
**Page S9**	**Figure 7.** Flow cytometry analysis of mouse heart cells at 2 days post-treatment. Live single cells were sorted into leukocyte subsets based on the expression of CD45, CD11b, Ly6G, CD3, B220, F480 and Ly6C.

Page S10 **Figure 8.** Flow cytometry analysis of mouse spleen cells at 2 days post-treatment. Live single cells were sorted into leukocyte subsets based on the expression of CD45, CD11b, Ly6G, CD3, B220, F480 and Ly6C.

Page S11 **Figure 9.** The number of adherent macrophages after culture on rHC matrices.

Page S12-13 **Figure 10.** Myocardial strain is improved in rHCI mice two days post-injection within the mid anterior LV wall which is the targeted injection region.

Page S14 **Figure 11.** Schematic depiction for the definition of the different histological areas within the infarcted myocardium.

Page S15 **Figure 12.** Gating strategies used in this work.

Page S16 **Table 1.** ECG analysis.

Page S16 **Table 2.** List of qPCR primers.

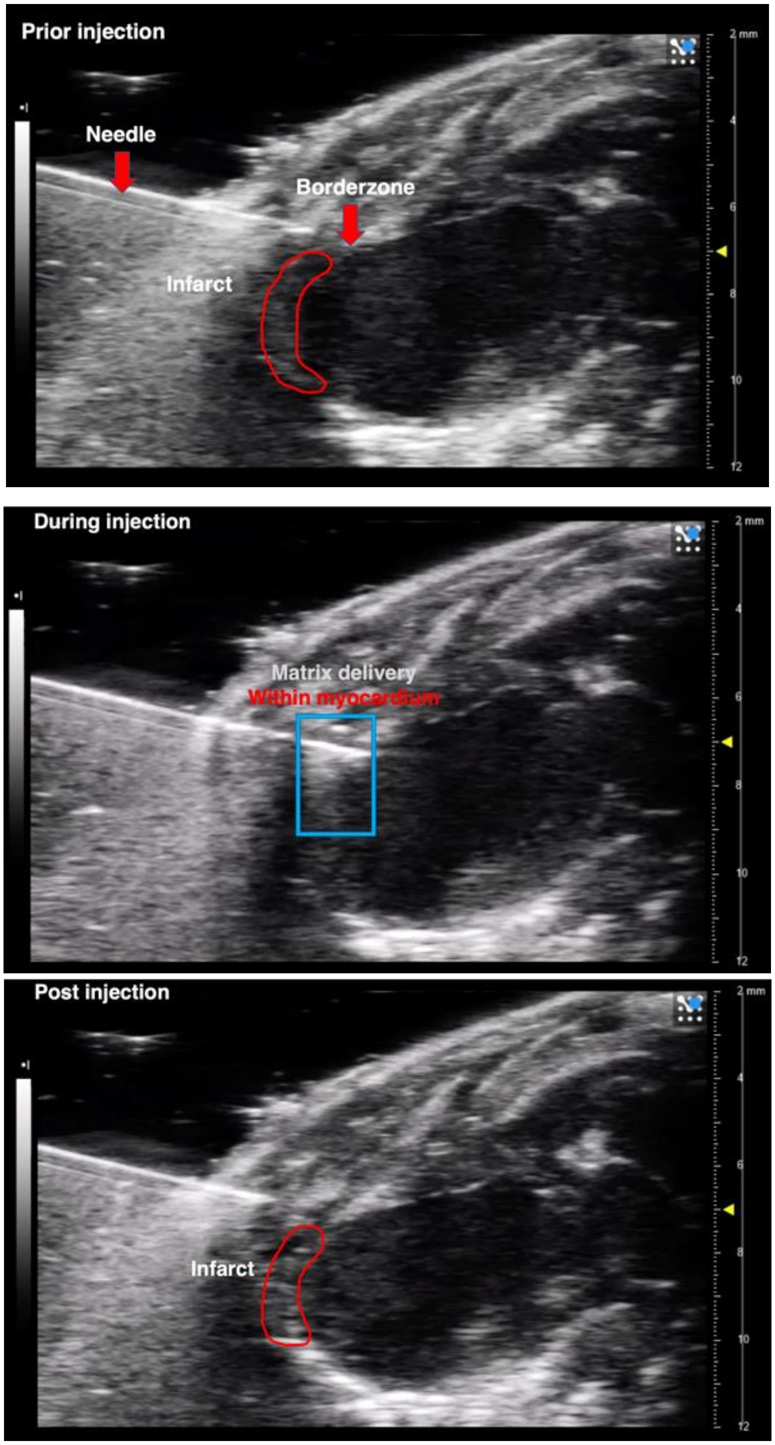

Fig. 1. Representative images depicting the intramyocardial injections for the rHCI matrix. Images shown are at time-points prior to intramyocardial delivery (top), during the injection (middle), and post-injection (bottom). Images were captured from a video recorded using a Vevo 3100 ultrasound system operating at 30 MHz. The video is available in the video supplementary information section of this article.

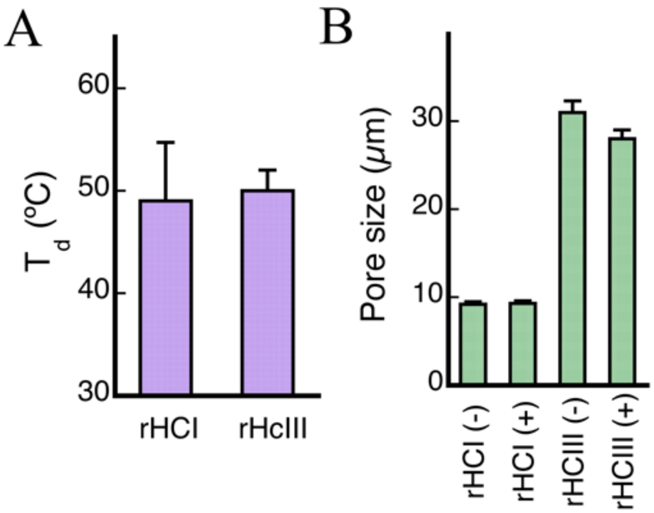

Fig. 2. Effect of chondroitin sulfate on the collagen matrices' physical properties. (A) Denaturation temperatures for collagen matrices prepared without chondroitin sulfate (T_d) (ºC; n=3). Bars represent mean ± SEM. (B) Mean pore size measured for collagen matrices with (+) or without (-) chondroitin sulfate. Bars represent mean ± SD. For A-B, n indicates the number of hydrogel batches.

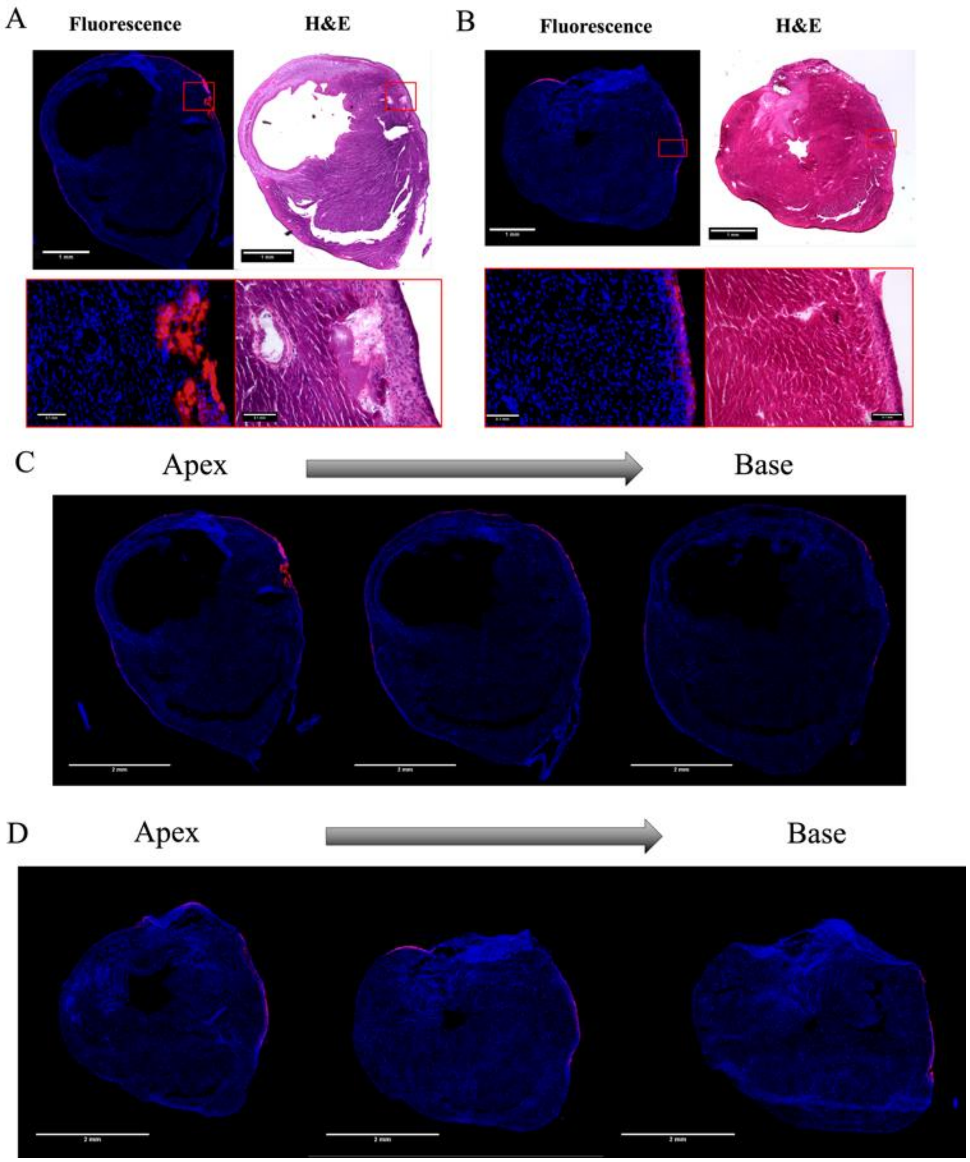

Fig. 3. Histological examination of rHC hydrogel-treated MI mouse hearts at 2 hours post-injection. The sections show fluorescently labeled (A) rHCI hydrogel and (B) rHCIII hydrogel. Fluorescence images show AF594 labeled rHCI/III hydrogels (red) and DAPI-stained cell nuclei (blue). Corresponding serial sections were stained with H&E. Scale bars for top images = 1 mm. Red boxes in the top images identify the area that is magnified below (scale bars = 0.1 mm). (C) and (D) depict fluorescence images of sections at different depths for the hearts that received the AF594 labeled (C) rHCI or (D) rHCIII matrices. The sections start at around 1.44 mm from the Apex (denoted as Apex) and increase in 0.48 mm increments towards the base of the heart (aorta/valve). Scale bars = 2 mm.

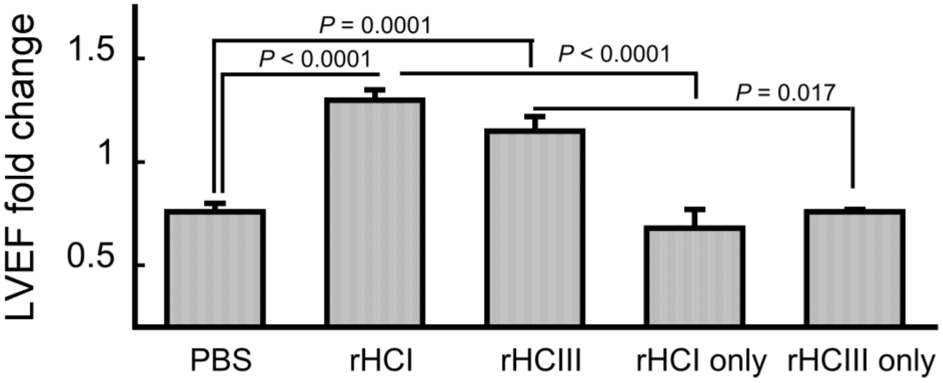

Fig. 4. Changes in left ventricle ejection fraction (LVEF%) for collagen matrices (rHCI and rHCIII) and non-crosslinked collagen solutions (rHCI only and rHCIII only). Sample sizes for rHCI only and rHCIII only were n=3-5. P values were calculated from a one-way ANOVA for effect of treatment followed by Holm's corrected multiple comparisons. The n values indicate the number of mice per group.

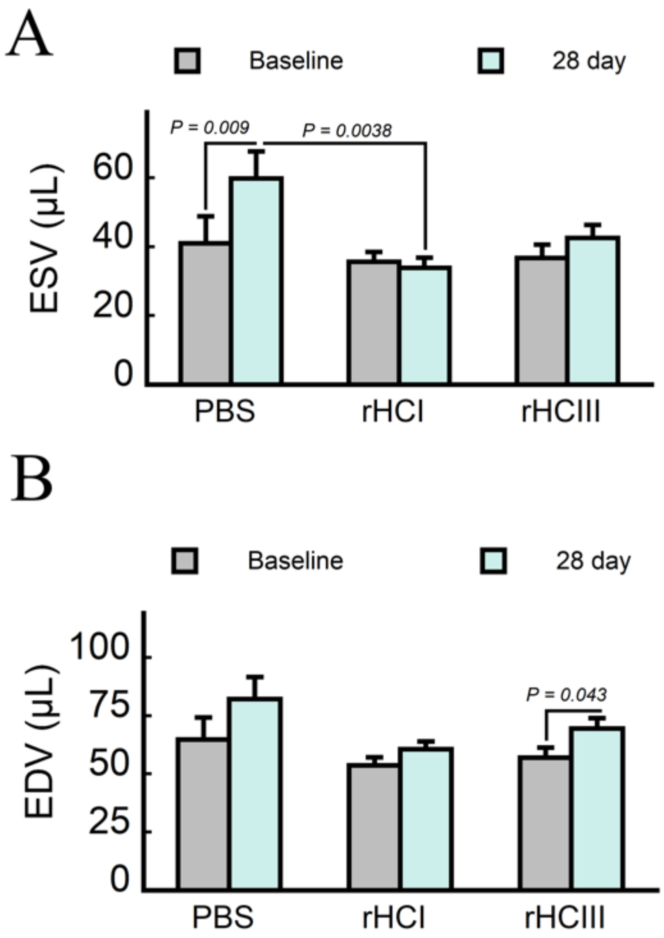

Fig. 5. Change in (A) end-systolic volume (ESV) and (B) end-diastolic volume (EDV) for the different treatment groups. Bars represent mean ± SEM (n=11 for PBS, n=15 for rHCI, and n=13 for rHCIII). P values were calculated from a two way ANOVA followed by Holm's corrected multiple comparisons. For A-B, n indicates the number of mice per group.

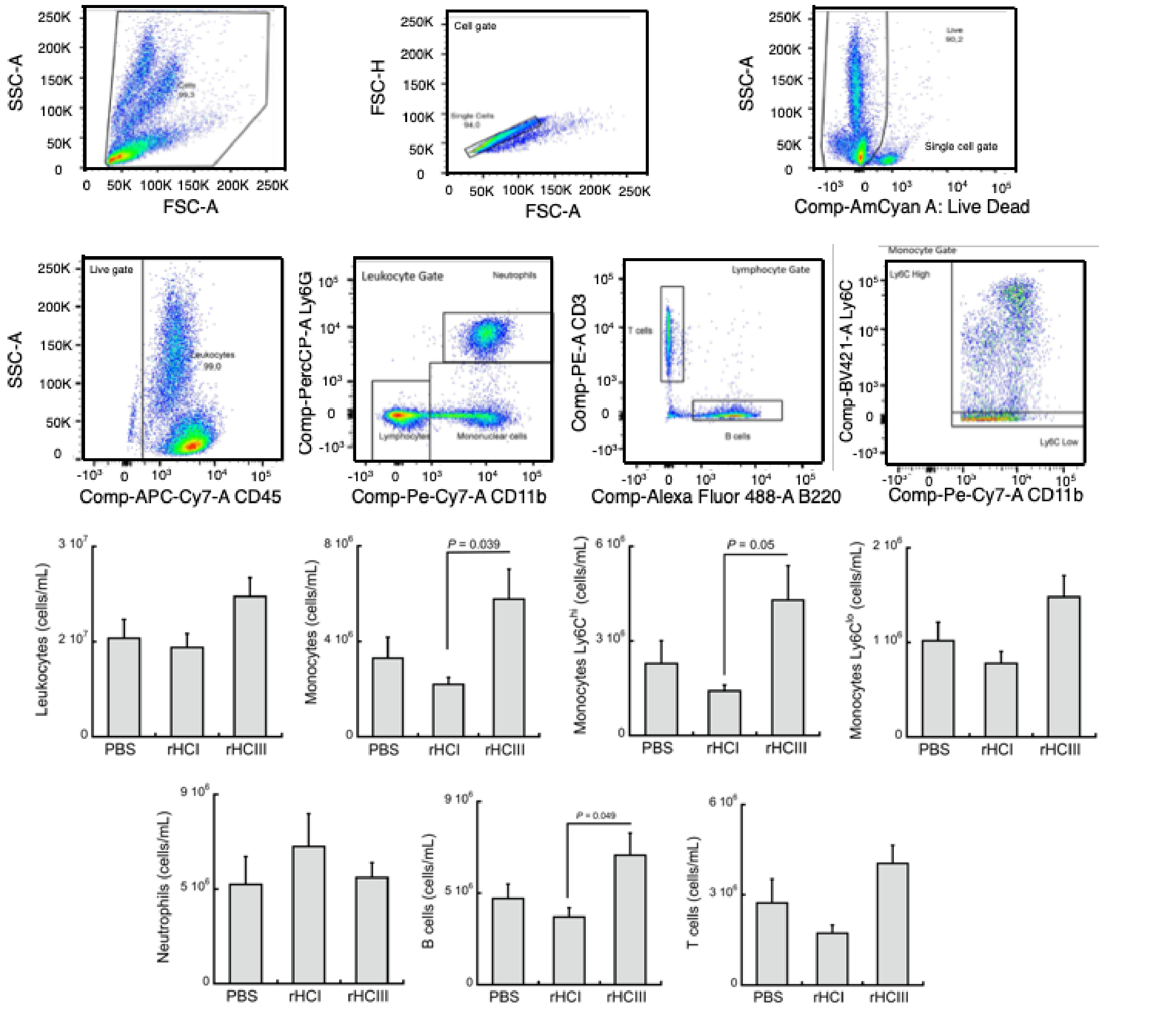

Fig. 6. Flow cytometry analysis of mouse blood at 2 days post-treatment. Live single cells were sorted into leukocyte subsets based on the expression of CD45, CD11b, Ly6G, CD3, B220, and Ly6C. P values were determined by a one-way ANOVA for effect of treatment followed by Holm’s correction for multiple comparisons. Data is represented as the mean ± SEM (n=5-7). The n values indicate the number of mice per group.

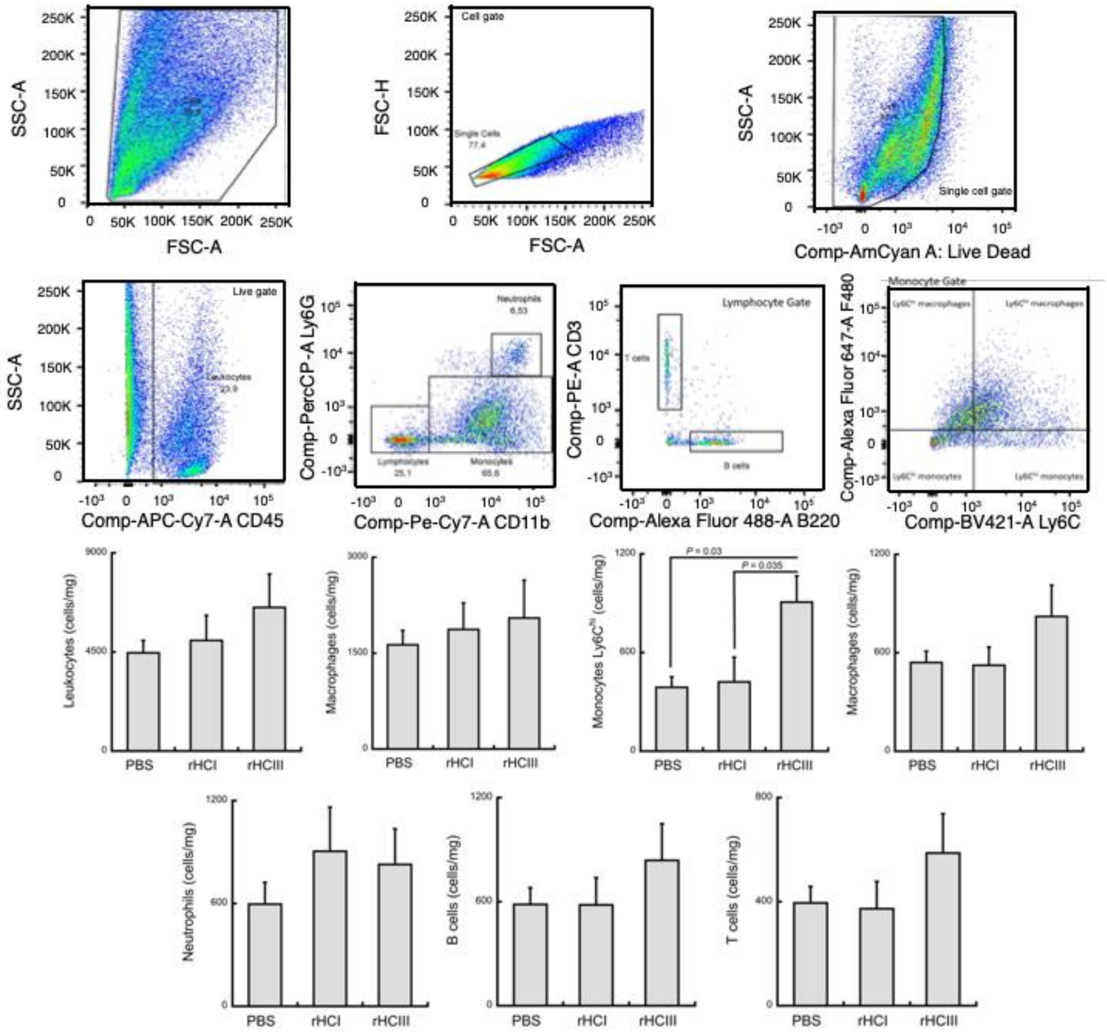

Fig. 7. Flow cytometry analysis of mouse heart cells at 2 days post-treatment. Live single cells were sorted into leukocyte subsets based on the expression of CD45, CD11b, Ly6G, CD3, B220, F480 and Ly6C. P values were determined by a one-way ANOVA for effect of treatment followed by Holm's correction for multiple comparisons. Data is represented as the mean ±SEM (n=5-7). The n values indicate the number of mice per group.

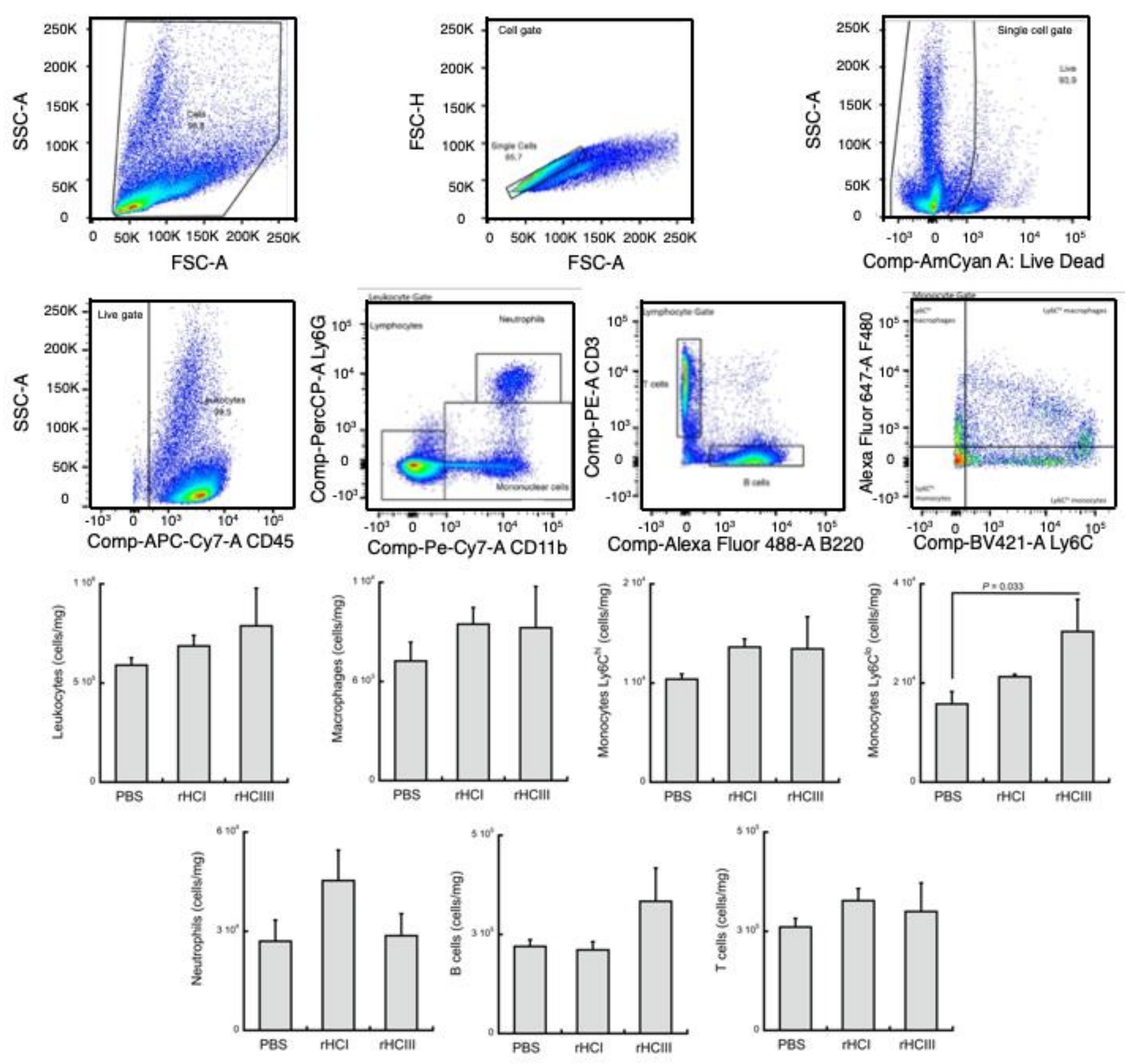

Fig. 8. Flow cytometry analysis of mouse spleen cells at 2 days post-treatment. Live single cells were sorted into leukocyte subsets based on the expression of CD45, CD11b, Ly6G, CD3, B220, F480 and Ly6C. P values were determined by a one-way ANOVA for effect of treatment followed by Holm's correction for multiple comparisons. Data is represented as the mean ± SEM (n=5-7). The n values indicate the number of mice per group.

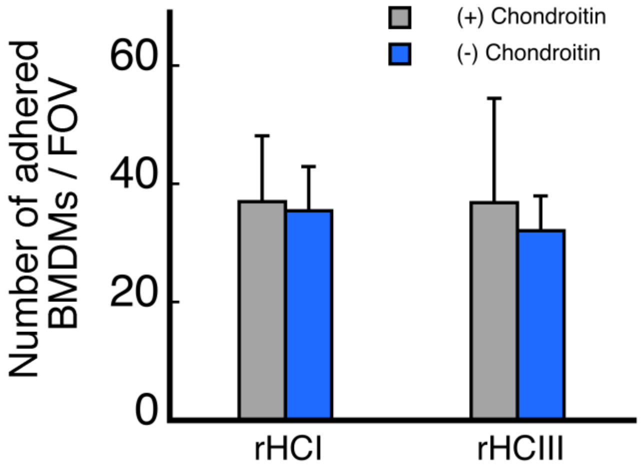

Fig. 9. Number of adherent macrophages after culture on rHC matrices (n=4). Bars represent mean ± SD. n indicates the number of mice (mouse bone marrow) per group.

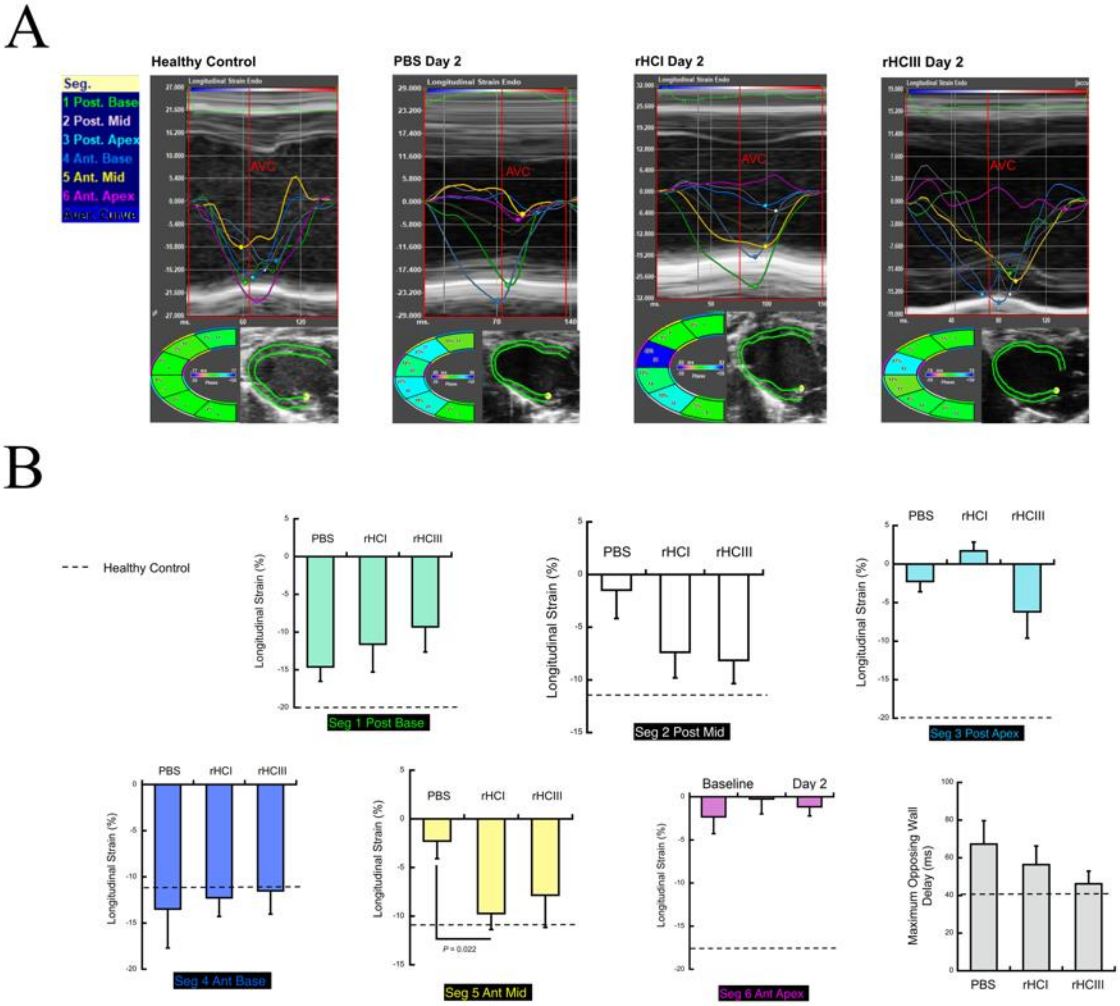

Fig. 10. Myocardial strain is improved in rHCI mice two days post-injection within the mid anterior LV wall which is the targeted injection region. (A) Example images from Vevostrain analysis of long axis B mode echocardiograms taken at two days post-injection in mice which were treated seven days post-MI. A healthy aged matched control in addition to PBS and rHCI treated mice are shown. For the strain analysis, the LV is split into six segments indicated in the legend. The region where the treatments are injected is highlighted (seg 5 anterior mid). The strain curves in each segment indicate the displacement of the endocardium over time through the cardiac contractile cycle. Strain is reported as a percentage indicating the change in position of the endocardium relative to its initial position at end diastole. The heart shortens in the longitudinal direction as it contracts, therefore the strain will become more negative during systole and in a healthy animal should peak at the aortic valve closure (AVC). To determine the impact of rHCI injection on cardiac contractility the strain reached at the time of the AVC (red line) was used for analysis. Below the strain curves is an example of the segmentation beside the echocardiogram of the heart. The values within the segmentation diagram correspond to the peak percent strain achieved in that region along with the phase of the cardiac cycle

when that strain was reached. (B) Data are presented as mean ± SEM for longitudinal strain at the time of aortic valve closure within each segment of the LV. The final graph indicates the maximum opposing wall delay which is the difference in the time to reach peak strain between anterior and posterior regions of the LV myocardium. PBS (n=12), rHCI (n=11), rHCII (n=5). Significance was determined with a one-way ANOVA for effect of treatment followed up with Holm-corrected post-hoc tests between treatment groups. The dashed line indicates the average value of aged matched healthy control mice. The n value indicates the number of mice per group.

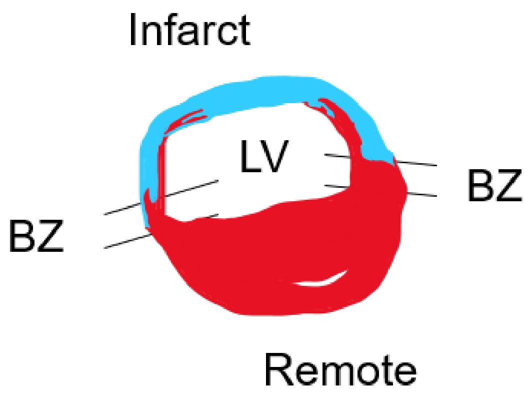

Fig. 11. Schematic depiction for the definition of the different histological areas within the infarcted myocardium. BZ: Border zone, LV: Left ventricle lumen.

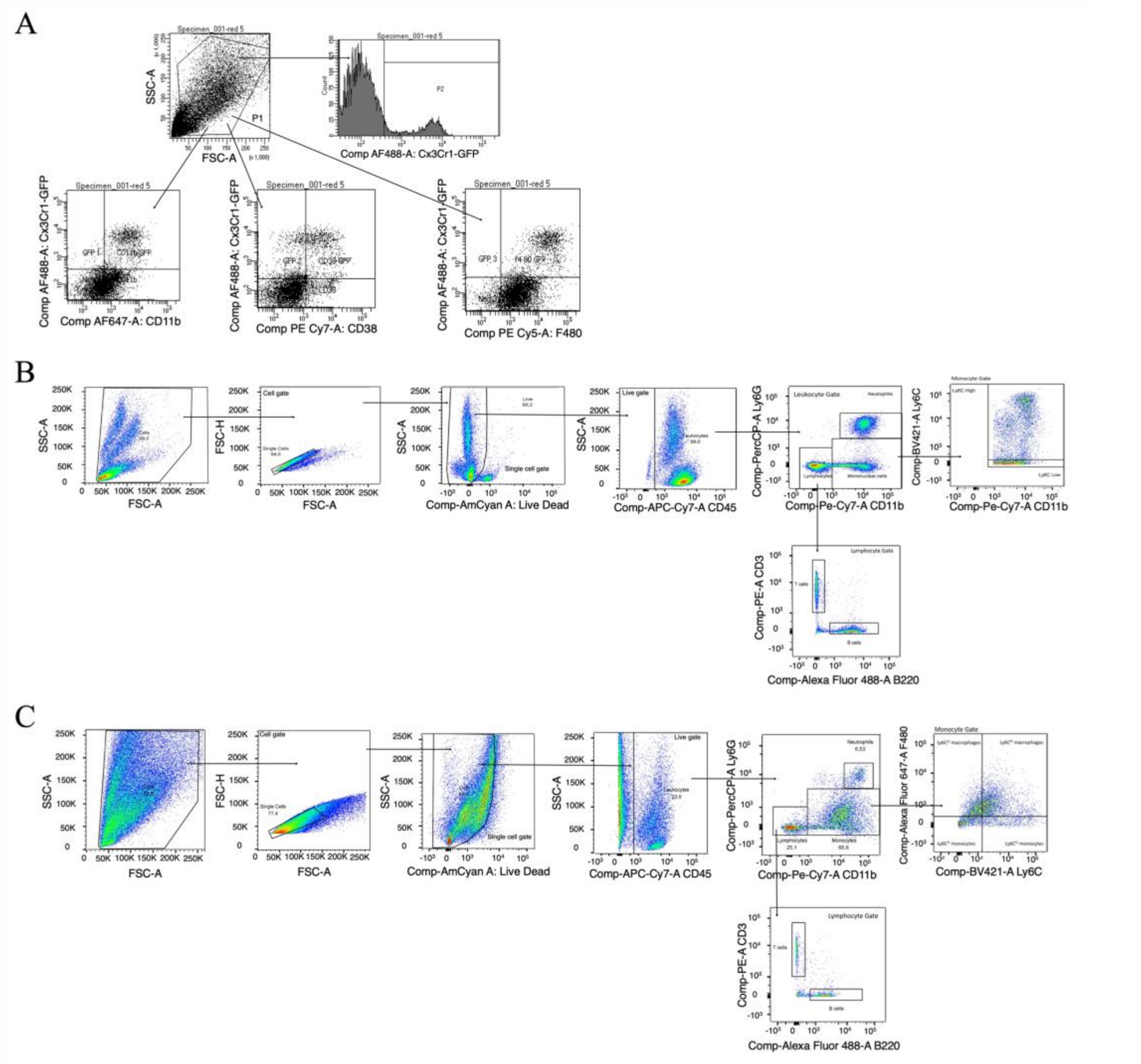

Fig. 12. Gating strategies used in this work. (A) Gating strategy for identification of Cx3Cr1-GFP$^+$ cells in the hearts of *Cx3Cr1*^{GFP} mice. This gating strategy also shows how Cx3Cr1-GFP$^+$ CDllb$^+$, Cx3Cr1-GFP$^+$ CD38$^+$ and Cx3Cr1-GFP$^+$ F480$^+$ sub-populations were identified. (B) Gating strategy to identify inflammatory cells in the blood two days post-treatment injection in a C57Bl/6 mouse myocardial infarction (MI) model. The following populations were analyzed: CD45$^+$ leukocytes, CD45$^+$CD11b$^+$Ly6G$^-$ monocytes, CD45$^+$CD11b$^+$Ly6G$^-$Ly6C$^+$ monocytes, CD45$^+$CD11b$^+$Ly6G$^-$Ly6C$^-$ monocytes, CD45$^+$CD11b$^+$Ly6G$^+$ neutrophils, CD45$^+$CD11b$^-$Ly6G$^-$CD3$^+$B220$^-$ T cells and CD45$^+$CD11b$^-$Ly6G$^-$B220$^+$CD3$^-$ B cells. (C) Gating strategy to identify inflammatory cells in the heart and spleen two days post-treatment injection in a C57Bl/6 mouse myocardial infarction (MI) model. The following populations were analyzed: CD45$^+$ leukocytes, CD45$^+$CD11b$^+$Ly6G$^-$ monocytes, CD45$^+$CD11b$^+$Ly6G$^-$F480$^-$Ly6C$^+$ monocytes, CD45$^+$CD11b$^+$Ly6G$^-$ F480$^-$Ly6C$^-$ monocytes, CD45$^+$CD11b$^+$Ly6G$^-$ F480$^+$ macrophages, CD45$^+$CD11b$^+$Ly6G$^+$ neutrophils, CD45$^+$CD11b$^-$Ly6G$^-$CD3$^+$B220$^-$ T cells and CD45$^+$CD11b$^-$Ly6G$^-$B220$^+$CD3$^-$ B cells.

Group	*n*	*Heart rate*	*PR interval*	*QRS interval*	*QT interval*	*QT (%)*
PBS	11	13.6±20.2	-0.4±28.1	2.3±27.9	-7.0±24.3	5.5±32.9
rHCI	12	22.0±10.6	-36.7±31.4 *	-12.3±22.6	-14.6±28.5	4.3±37.1
rHCIII	5	16.6±12.0	16.7±18.1	9.8±21.7	9.4±15.0	27.7±22.1

*$p<0.05$, vs PBS and rHCIII, calculated from Tukey's HSD test

Table 1. ECG-derived parameters measured at 2 days post-treatment. Values represented as % change to baseline.

Gene	Forward	Reverse
α-SMA	GCCCAGCTTCGTCGTATTCC	ACGGCCGCCTCCTCTTCCTC
MMP-1	GTG CTC TCC TTCCACAGAGG	GGTCCACGTCTCATCAAGGT
MMP-2	AAGGATGGACTCCTGGCACATGCCTTT	ACCTGTGGGCTTGTCACGTGGTGT
MMP-9	AAGGACGGCCTTCTGGCACACGCCTTT	GTGGTATAGTGGGACACATAGTGG
TIMP2	CGTTTTGCAATGCAGACGTA	GAATCCTCTTGATGGGGTTG
TIMP1	GTAAGGCCTGTAGCTGTGCC	AGGTGGTCTCGTTGATTCGT
Arg1	AAAGCTGGTCTGCTGGAAAA	ACAGACCGTGGGTTCTTCAC
18S	AAACGGCTACCACATCCAAG	CCTCCAATGGATCCTCGTTA

Table 2. List of qPCR primers.
