# Supplementary material for: Injectable human recombinant collagen matrices limit adverse remodeling and improve cardiac function after myocardial infarction
Source: Nat Commun. 2019 Oct 25;10:4866. doi: 10.1038/s41467-019-12748-8 (PMC6814728; doi:10.1038/s41467-019-12748-8)
Supplement: Supplementary file 3 — Reporting Summary [file 41467_2019_12748_MOESM3_ESM.pdf]

nature research

Corresponding author(s): Emilio I. Alarcon

Last updated by author(s): Sep 17, 2019

Reporting Summary

Nature Research wishes to improve the reproducibility of the work that we publish. This form provides structure for consistency and transparency in reporting. For further information on Nature Research policies, see Authors & Referees and the Editorial Policy Checklist .

Statistics

For all statistical analyses, confirm that the following items are present in the figure legend, table legend, main text, or Methods section.

n/a	Confirmed	
☐	☒	The exact sample size (*n*) for each experimental group/condition, given as a discrete number and unit of measurement
☒	☐	A statement on whether measurements were taken from distinct samples or whether the same sample was measured repeatedly
☐	☒	The statistical test(s) used AND whether they are one- or two-sided<br>*Only common tests should be described solely by name; describe more complex techniques in the Methods section.*
☐	☒	A description of all covariates tested
☐	☒	A description of any assumptions or corrections, such as tests of normality and adjustment for multiple comparisons
☐	☒	A full description of the statistical parameters including central tendency (e.g. means) or other basic estimates (e.g. regression coefficient) AND variation (e.g. standard deviation) or associated estimates of uncertainty (e.g. confidence intervals)
☐	☒	For null hypothesis testing, the test statistic (e.g. *F*, *t*, *r*) with confidence intervals, effect sizes, degrees of freedom and *P* value noted<br>*Give P values as exact values whenever suitable.*
☒	☐	For Bayesian analysis, information on the choice of priors and Markov chain Monte Carlo settings
☒	☐	For hierarchical and complex designs, identification of the appropriate level for tests and full reporting of outcomes
☒	☐	Estimates of effect sizes (e.g. Cohen's *d*, Pearson's *r*), indicating how they were calculated

Our web collection on statistics for biologists contains articles on many of the points above.

Software and code

Policy information about availability of computer code

Data collection	Flow cytometry: BD FACS Diva (v8.0.1) Young's modulus: Instron Series IX/S Fluorescent Images: Zen Blue v2.6 Brightfield Images: Infinity Capture Echocardiography: Vevo 770 (LV functional measurements), Vevo 3100 (for strain analysis)
Data analysis	Statistical analysis: Kaleida Graph 4.5®, STATA/IC 15.1 Echocardiography: Vevo 770 V3.0.0., Vevo LAB 3.1.1. with VevoStrain Histology and ICC: MIQuant and Fiji Flow Cytometry: Flowjo v10, BD FACS Diva (v8.0.1)

For manuscripts utilizing custom algorithms or software that are central to the research but not yet described in published literature, software must be made available to editors/reviewers. We strongly encourage code deposition in a community repository (e.g. GitHub). See the Nature Research guidelines for submitting code & software for further information.

Data

Policy information about availability of data

All manuscripts must include a data availability statement. This statement should provide the following information, where applicable:

- Accession codes, unique identifiers, or web links for publicly available datasets
- A list of figures that have associated raw data
- A description of any restrictions on data availability

All data needed to evaluate the conclusions are present in the manuscript and Supplementary Materials. Materials or additional information related to this study may be requested from the authors. We have prepared individual folders for each figure that includes raw data and statistical analysis.

Field-specific reporting

Please select the one below that is the best fit for your research. If you are not sure, read the appropriate sections before making your selection.

Life sciences study design

All studies must disclose on these points even when the disclosure is negative.

Sample size	11-15 for in vivo experiments (Fig. 2) Sample size was determined based on previous experience with this model.
Data exclusions	Animals which did not successfully sustain an infarction post-surgery and those where the injections were suboptimal were excluded from the study. Further details are found in the raw data analysis files for Fig. 2. No data was excluded following analysis.
Replication	NA
Randomization	Animals were randomly selected for the treatments.
Blinding	Animals received the injections in a blinded-fashion as the researcher injecting the animal was not told of the treatment being given to the animal. Further, the data was also analyzed in a blinded-fashion.

Reporting for specific materials, systems and methods

We require information from authors about some types of materials, experimental systems and methods used in many studies. Here, indicate whether each material, system or method listed is relevant to your study. If you are not sure if a list item applies to your research, read the appropriate section before selecting a response.

Materials & experimental systems

n/a	Involved in the study
☐	☒ Antibodies
☐	☒ Eukaryotic cell lines
☐	☐ Palaeontology
☐	☒ Animals and other organisms
☐	☐ Human research participants
☐	☐ Clinical data

Methods

n/a	Involved in the study
☐	☐ ChIP-seq
☐	☒ Flow cytometry
☐	☐ MRI-based neuroimaging

Antibodies

Antibodies used

Flow Cytometry Mouse Antibodies:

Mouse monocyte subset analysis-
CD45-APC/Fire 750 (30-F11), 1:160, Cat# 103154
CD11b-PE/Cy7 (M1/70), 1:80, Cat#101216
Ly6G-PerCP/Cy5.5 (1A8), 1:160, Cat#127615
CD3-PE (17A2), 1:160, Cat#100205
B220-AF488 (RA3-6B2), 1:50, Cat#103228
F4/80-AF647 (BM8), 1:100, Cat#123122
Ly6C-BV421 (AL-21), 1:80, Cat#562727 BD Biosciences
Cx3cr1-EGFP experiment-
CD11b-APC (M1/70), 1:80, Cat#101211
Ly6G/6C-PE (RB6-8C5), 1:80, Cat#108407
F4/80-PE/Cy5 (BM8), 1:20, Cat#123111
CD38-PE/Cy7 (90), 1:80, Cat#102717
Cd206-AF700 (C068C2), 1:200, Cat#141733
All from Biolegend unless otherwise indicated.

IHC for frozen mouse heart sections:
PECAM-1 (RM0032-1D12), 1:50, Cat#101454 Santa Cruz
α-Smooth muscle actin, 1:200, Cat#ab5694 Abcam
CD206-AF488 (C068C2), 1:50, Cat#141710 Biolegend
Cardiac Troponin I, 1:200, Cat#ab56357 Abcam
Cardiac Troponin I, 1:200, Cat#ab188877 Abcam
Connexin 43/GJA1, 1:400, Cat#ab11370 Abcam
ICC rat neonatal cardiomyocytes:
α-Sarcomeric actinin, 1:400, Cat#A7811, Sigma
Connexin 43 1:200, Cat#C6219, Sigma

	α5 integrin 1:250, Cat#ab150361, Abcam
Validation	Antibodies used in this study were optimized for their use. The endpoints for optimal dilutions depended the assay (histology vs. flow cytometry). Dilutions used in our study are described in our manuscript, and further experimental protocols can be obtained directly from our team.

Eukaryotic cell lines

Policy information about cell lines

Cell line source(s)	Neonatal rat ventricular myocytes (NRVMs) Sprague–Dawley rats and bone marrow-derived macrophages C57BL/6J mice. Those cells were isolated freshly in our laboratories.
Authentication	NVRMs were isolated following a protocol described in J Physiol 593, 1147-1157 (2015), Dr. Liang is an expert on this protocol. As per the macrophages, those were isolated following a protocol described in CSH protocols 2008, pdb.prot5080 (2008).
Mycoplasma contamination	Cells were not tested for mycoplasma contamination since those were used freshly after isolation.
Commonly misidentified lines (See ICLAC register)	N/A

Palaeontology

Specimen provenance	*Provide provenance information for specimens and describe permits that were obtained for the work (including the name of the issuing authority, the date of issue, and any identifying information).*
Specimen deposition	*Indicate where the specimens have been deposited to permit free access by other researchers.*
Dating methods	*If new dates are provided, describe how they were obtained (e.g. collection, storage, sample pretreatment and measurement), where they were obtained (i.e. lab name), the calibration program and the protocol for quality assurance OR state that no new dates are provided.*

☐ Tick this box to confirm that the raw and calibrated dates are available in the paper or in Supplementary Information.

Animals and other organisms

Policy information about studies involving animals; ARRIVE guidelines recommended for reporting animal research

Laboratory animals	C57BL/6 mice (Charles River), and B6.129P-Cx3cr1 tm1Litt/J mice (Jackson Laboratory)
Wild animals	*Provide details on animals observed in or captured in the field; report species, sex and age where possible. Describe how animals were caught and transported and what happened to captive animals after the study (if killed, explain why and describe method; if released, say where and when) OR state that the study did not involve wild animals.*
Field-collected samples	*For laboratory work with field-collected samples, describe all relevant parameters such as housing, maintenance, temperature, photoperiod and end-of-experiment protocol OR state that the study did not involve samples collected from the field.*
Ethics oversight	All procedures were approved by the University of Ottawa Animal Care Committee, and performed according to the National Institute of Health Guide for the Care and Use of Laboratory Animals

Note that full information on the approval of the study protocol must also be provided in the manuscript.

Human research participants

Policy information about studies involving human research participants

Population characteristics	*Describe the covariate-relevant population characteristics of the human research participants (e.g. age, gender, genotypic information, past and current diagnosis and treatment categories). If you filled out the behavioural & social sciences study design questions and have nothing to add here, write "See above."*
Recruitment	*Describe how participants were recruited. Outline any potential self-selection bias or other biases that may be present and how these are likely to impact results.*
Ethics oversight	*Identify the organization(s) that approved the study protocol.*

Note that full information on the approval of the study protocol must also be provided in the manuscript.

Clinical data

Policy information about clinical studies

All manuscripts should comply with the ICMJE guidelines for publication of clinical research and a completed CONSORT checklist must be included with all submissions.

Clinical trial registration	*Provide the trial registration number from ClinicalTrials.gov or an equivalent agency.*
Study protocol	*Note where the full trial protocol can be accessed OR if not available, explain why.*
Data collection	*Describe the settings and locales of data collection, noting the time periods of recruitment and data collection.*
Outcomes	*Describe how you pre-defined primary and secondary outcome measures and how you assessed these measures.*

ChIP-seq

Data deposition

☐ Confirm that both raw and final processed data have been deposited in a public database such as GEO.

☐ Confirm that you have deposited or provided access to graph files (e.g. BED files) for the called peaks.

Data access links<br>*May remain private before publication.*	*For "Initial submission" or "Revised version" documents, provide reviewer access links. For your "Final submission" document, provide a link to the deposited data.*
Files in database submission	*Provide a list of all files available in the database submission.*
Genome browser session<br>(e.g. UCSC)	*Provide a link to an anonymized genome browser session for "Initial submission" and "Revised version" documents only, to enable peer review. Write "no longer applicable" for "Final submission" documents.*

Methodology

Replicates	*Describe the experimental replicates, specifying number, type and replicate agreement.*
Sequencing depth	*Describe the sequencing depth for each experiment, providing the total number of reads, uniquely mapped reads, length of reads and whether they were paired- or single-end.*
Antibodies	*Describe the antibodies used for the ChIP-seq experiments; as applicable, provide supplier name, catalog number, clone name, and lot number.*
Peak calling parameters	*Specify the command line program and parameters used for read mapping and peak calling, including the ChIP, control and index files used.*
Data quality	*Describe the methods used to ensure data quality in full detail, including how many peaks are at FDR 5% and above 5-fold enrichment.*
Software	*Describe the software used to collect and analyze the ChIP-seq data. For custom code that has been deposited into a community repository, provide accession details.*

Flow Cytometry

Plots

Confirm that:

☒ The axis labels state the marker and fluorochrome used (e.g. CD4-FITC).

☒ The axis scales are clearly visible. Include numbers along axes only for bottom left plot of group (a 'group' is an analysis of identical markers).

☒ All plots are contour plots with outliers or pseudocolor plots.

☒ A numerical value for number of cells or percentage (with statistics) is provided.

Methodology

Sample preparation	Monocyte subset experiment: Two days post-treatment injection mice were sacrificed by CO_2 inhalation followed by cervical dislocation. Blood was collected from mice through cardiac puncture in a 50 mM EDTA solution and RBCs were lysed with red blood cell (RBC) lysis buffer according to the manufacturer's protocol (Biolegend 420301). Hearts were perfused with PBS and cells were isolated from harvested mouse hearts using a digestion buffer containing: DNAse I (50 U/µL; Sigma D5025), collagenase type II (400 U/mL; ThermoFisher 17101015), collagenase D (0.15 U/mL; Sigma 11088866001) and hyaluronidase (10 U/mL; Sigma H3506). Following an hour digestion at 37ºC, isolated heart cells were passed through a 70 µm filter. Finally, harvested mouse spleens were mashed and triturated through a 70 µm filter followed by incubation with red blood cell (RBC) lysis buffer. Cell pellets were collected by centrifugation at 400xg for 5 min at 4◦C. Isolated cells from all tissues were incubated

with Zombie Aqua fixable viability dye (1:500, Biolegend 423101) for 20 min at room temperature. Next, Fc receptors were blocked with TruStain X reagent (1:100, Biolegend 103319) for 10 min at room temperatue. The cells were then incubated with an antibody cocktail for 45 min at room temperature containing CD45-APC/Fire 750 , CD11b-PE/Cy7, Ly6G-PerCP/Cy5.5, CD3-PE, B220-AF488, F480-AF647 and Ly6C-BV421.
Cx3cr1 Experiment:
To evaluate the recruitment of circulating mononuclear cells to the myocardium following rHC treatment, B6.129P-Cx3cr1 tm1Litt/J mice (Cx3cr1-EGFP) were purchased from the Jackson Laboratory and used as previously described 61. Briefly, at 1 week post-MI, mice received treatment of 50µl of PBS, rHCI or rHCIII, as described above. Animals were sacrificed 2 days after treatment and hearts were perfused with PBS and the right ventricle and the apical region of the left ventricle were collected. The tissues were rinsed with HBSS and digested in 2.4U/ml dispase I (Roche) and 1mg/ml Collagenase B (Roche) for 40 min at 37° C. Samples were washed 3 times with PBS, centrifuged for 5 min at 400g, and the isolated cells were prepared for flow cytometry . Cells were labeled with APC anti-mouse/human CD11b, PE anti-mouse Ly-6G/6C, PE/Cy5 anti-mouse F4/80, PE/Cy7 anti-mouse CD38 and Alexa Fluor® 700 anti-mouse CD206, following the manufacturer-recommended dilutions.

Instrument: BD FACS Aria III

Software: FlowJo software v10 and BDFACS Diva v8.0.1

Cell population abundance: Representative examples for cell abundance are found in Figs. 6-8

Gating strategy: Representative examples for the gating strategy used are found in Supplementary Figs. 6-8. Further details on the specific gating strategies can be obtained from the authors.

☒ Tick this box to confirm that a figure exemplifying the gating strategy is provided in the Supplementary Information.

Magnetic resonance imaging

Experimental design

Design type: *Indicate task or resting state; event-related or block design.*

Design specifications: *Specify the number of blocks, trials or experimental units per session and/or subject, and specify the length of each trial or block (if trials are blocked) and interval between trials.*

Behavioral performance measures: *State number and/or type of variables recorded (e.g. correct button press, response time) and what statistics were used to establish that the subjects were performing the task as expected (e.g. mean, range, and/or standard deviation across subjects).*

Acquisition

Imaging type(s): *Specify: functional, structural, diffusion, perfusion.*

Field strength: *Specify in Tesla*

Sequence & imaging parameters: *Specify the pulse sequence type (gradient echo, spin echo, etc.), imaging type (EPI, spiral, etc.), field of view, matrix size, slice thickness, orientation and TE/TR/flip angle.*

Area of acquisition: *State whether a whole brain scan was used OR define the area of acquisition, describing how the region was determined.*

Diffusion MRI ☐ Used ☐ Not used

Preprocessing

Preprocessing software: *Provide detail on software version and revision number and on specific parameters (model/functions, brain extraction, segmentation, smoothing kernel size, etc.).*

Normalization: *If data were normalized/standardized, describe the approach(es): specify linear or non-linear and define image types used for transformation OR indicate that data were not normalized and explain rationale for lack of normalization.*

Normalization template: *Describe the template used for normalization/transformation, specifying subject space or group standardized space (e.g. original Talairach, MNI305, ICBM152) OR indicate that the data were not normalized.*

Noise and artifact removal: *Describe your procedure(s) for artifact and structured noise removal, specifying motion parameters, tissue signals and physiological signals (heart rate, respiration).*

Volume censoring: *Define your software and/or method and criteria for volume censoring, and state the extent of such censoring.*

Statistical modeling & inference

Model type and settings: *Specify type (mass univariate, multivariate, RSA, predictive, etc.) and describe essential details of the model at the first and second levels (e.g. fixed, random or mixed effects; drift or auto-correlation).*

Effect(s) tested	*Define precise effect in terms of the task or stimulus conditions instead of psychological concepts and indicate whether ANOVA or factorial designs were used.*

Specify type of analysis: ☐ Whole brain ☐ ROI-based ☐ Both

Statistic type for inference<br>(See Eklund et al. 2016)	*Specify voxel-wise or cluster-wise and report all relevant parameters for cluster-wise methods.*
Correction	*Describe the type of correction and how it is obtained for multiple comparisons (e.g. FWE, FDR, permutation or Monte Carlo).*

Models & analysis

n/a	Involved in the study
☐	☐ Functional and/or effective connectivity
☐	☐ Graph analysis
☐	☐ Multivariate modeling or predictive analysis

Functional and/or effective connectivity	*Report the measures of dependence used and the model details (e.g. Pearson correlation, partial correlation, mutual information).*
Graph analysis	*Report the dependent variable and connectivity measure, specifying weighted graph or binarized graph, subject- or group-level, and the global and/or node summaries used (e.g. clustering coefficient, efficiency, etc.).*
Multivariate modeling and predictive analysis	*Specify independent variables, features extraction and dimension reduction, model, training and evaluation metrics.*
