# Supplementary material for: Injectable human recombinant collagen matrices limit adverse remodeling and improve cardiac function after myocardial infarction
Source: Nat Commun. 2019 Oct 25;10:4866. doi: 10.1038/s41467-019-12748-8 (PMC6814728; doi:10.1038/s41467-019-12748-8)
Supplement: Supplementary file 4 — Description of Additional Supplementary Files [file 41467_2019_12748_MOESM4_ESM.pdf]

Title: Supplementary Movie 1.

Description: Representative echo-guided intramyocardial injections for the rHCl matrix.
